# Genome-Wide CRISPR/Cas9-Based Screening for Deubiquitinase Subfamily Identifies Ubiquitin-Specific Protease 11 as a Novel Regulator of Osteogenic Differentiation

**DOI:** 10.3390/ijms23020856

**Published:** 2022-01-13

**Authors:** Kamini Kaushal, Apoorvi Tyagi, Janardhan Keshav Karapurkar, Eun-Jung Kim, Parthasaradhireddy Tanguturi, Kye-Seong Kim, Han-Sung Jung, Suresh Ramakrishna

**Affiliations:** 1 Graduate School of Biomedical Science and Engineering, Hanyang University, Seoul 04763, Korea; kaminikaushal10@gmail.com (K.K.); apoorvityagi09@gmail.com (A.T.); kalpesh25021992@gmail.com (J.K.K.); parthabiochem7@gmail.com (P.T.); 2 Division in Anatomy and Developmental Biology, Department of Oral Biology, Taste Research Center, Oral Science Research Center, BK21 FOUR Project, Yonsei University College of Dentistry, Seoul 03722, Korea; BLUELEAH@yuhs.ac; 3 College of Medicine, Hanyang University, Seoul 04763, Korea

**Keywords:** protein degradation, ubiquitination, stem cells, regenerative medicine

## Abstract

The osteoblast differentiation capacity of mesenchymal stem cells must be tightly regulated, as inadequate bone mineralization can lead to osteoporosis, and excess bone formation can cause the heterotopic ossification of soft tissues. The balanced protein level of Msh homeobox 1 (MSX1) is critical during normal osteogenesis. To understand the factors that prevent MSX1 protein degradation, the identification of deubiquitinating enzymes (DUBs) for MSX1 is essential. In this study, we performed loss-of-function-based screening for DUBs regulating MSX1 protein levels using the CRISPR/Cas9 system. We identified ubiquitin-specific protease 11 (USP11) as a protein regulator of MSX1 and further demonstrated that USP11 interacts and prevents MSX1 protein degradation by its deubiquitinating activity. Overexpression of USP11 enhanced the expression of several osteogenic transcriptional factors in human mesenchymal stem cells (hMSCs). Additionally, differentiation studies revealed reduced calcification and alkaline phosphatase activity in USP11-depleted cells, while overexpression of USP11 enhanced the differentiation potential of hMSCs. These results indicate the novel role of USP11 during osteogenic differentiation and suggest USP11 as a potential target for bone regeneration.

## 1. Introduction

Homeoproteins are one of the major classes of transcriptional factors that regulate the development of tissues and organs in vertebrates [1]. The Msh homeobox 1 (MSX1) is a homeobox transcriptional factor required for the development of many organs, such as limbs, teeth, and the neural crest [2,3]. Elevated expression of Msx1 protein during early morphogenesis and initial skeletal patterning depicts the importance of Msx1 during early developmental stages [4]. Transgenic mice with alpha (I) collagen promoter-regulated Msx1 expression also showed an increased proportion of osteoblasts, including enhanced proliferation and apoptosis, suggesting the critical role of Msx1 in craniofacial bone modeling [5]. The controlled and timely expression of the *MSX1* gene is necessary for normal osteogenesis, craniofacial development, and limb-pattern formation [5,6,7]. Mutations in *Msx1* have been linked to craniofacial defects and tooth agenesis in mice [8,9], while genetic alterations in human *MSX1* are associated with cleft lip/palate and tooth agenesis [5,6,10,11,12]. Additionally, it was recently reported that Msx1-deficient mice exhibit alterations in craniofacial bones, including the premaxilla, maxilla, palatine bone, frontal bone, and mandible, as well as defects in the incisor and alveolar bone [13].

Proteins are effectors for most physiological functions in cells and play an important role in the extracellular medium. Protein function is fine-tuned at multiple levels, including protein synthesis and degradation, intracellular localization, and posttranslational modifications (PTMs). The PTM of proteins is an important mechanism for regulating their biological activity. PTMs such as SUMOylation, phosphorylation, and ubiquitination play critical roles in vital cellular processes, such as proliferation and differentiation in bone homeostasis [14,15,16,17]. A recent study reported that during embryonic limb development, the phosphorylation of Msx1 promotes cell proliferation through the Fibroblast growth factor 9/18- Mitogen-activated protein kinase (Fgf9/18-MAPK) signaling pathway [7]. In the past few years, extensive research has focused on elucidating the importance of the ubiquitin-proteasome system (UPS) in the regulation of osteogenesis and bone development [18,19]. Msx1 has been reported to undergo SUMO-1-mediated modifications to modulate its molecular functions during organogenesis [20]. A later study demonstrated that the protein inhibitor of activated STAT1 (PIAS1) negatively regulates the ubiquitination of MSX1 homeoprotein in a SUMO-independent manner [21]. Moreover, ubiquitination is a key process for maintaining the protein levels of any substrate by regulating its protein turnover [22,23]. The loss of function of any gene related to UPS results in the development of various diseases, including neurodegenerative disease, cancers, and osteoporosis [24,25,26,27,28].

Deubiquitination is a process where covalently attached ubiquitin molecules from the target protein are removed by deubiquitinating enzymes (DUBs). Ubiquitin-specific proteases (USPs) are the largest subfamily of DUBs involved in several cellular processes, including bone remodeling [17]. To date, extensive research has been carried out to investigate genetic aberration in the *MSX1* gene [29,30,31]. However, the molecular functions of MSX1 through its protein regulation by deubiquitination have not received much scrutiny. In this study, we focused on identifying DUBs for MSX1 using our recently developed CRISPR/Cas9-mediated DUB knockout library kit. Herein, we identified USP11, a deubiquitinase acting as a protein stabilizer for MSX1. Furthermore, the depletion of USP11 downregulated the expression of several osteogenic transcriptional factors in human mesenchymal stem cells (hMSCs). USP11 is critical for the efficient differentiation of hMSCs into the osteogenic lineage, suggesting the novel role of USP11 during osteogenic differentiation.

## 2. Results

### 2.1. CRISPR-Based Genome-Wide Screening for the DUBs Regulating MSX1 Protein Levels

**Figure 1:**
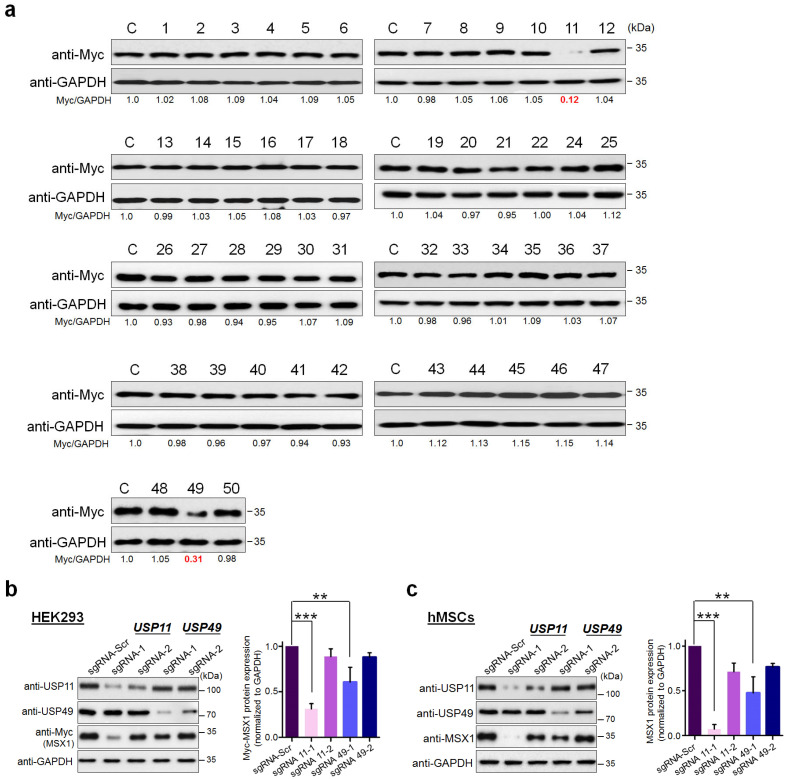
CRISPR-based genome-scale screening of USP sub-family proteins exhibiting a reduction in MSX1 protein levels. (**a**) Screening for DUBs regulating MSX1 was performed using a CRISPR/Cas9-based DUBKO sgRNA kit. HEK293 cells were transfected with Myc-MSX1 and the indicated sgRNAs along with Cas9. Equal concentrations of proteins from the sgRNA kit transfected HEK293 cells were subjected to Western blot analyses. The protein band intensities were estimated using ImageJ software with reference to the GAPDH control band for each individual sgRNA (Myc-MSX1/GAPDH). The loss of USPs leading to the downregulation of the MSX1 protein level is marked in red. (**b**) Myc-MSX1, sgRNAs targeting *USP11,* or sgRNAs targeting *USP49* were transfected in HEK293 cells. The protein band intensity of HEK293 cells transfected with sgRNA1 targeting *USP11* showed the highest reduction in MSX1. This experiment was performed in triplicates and band intensities were estimated using ImageJ software with reference to the GAPDH control band and are graphically represented. One-way ANOVA followed by Tukey’s post hoc test was used. Error bars represent ± SD, ** *p* < 0.001 and *** *p* < 0.0001. (**c**) The sgRNAs targeting *USP11* or sgRNAs targeting *USP49*, which potentially regulate endogenous MSX1 protein levels, were transfected in hMSCs along with Cas9. The protein band intensity of hMSCs transfected with sgRNA1 targeting *USP11* showed the highest reduction in MSX1. This experiment was performed in triplicates and band intensities were estimated using ImageJ software with reference to the GAPDH control band and are graphically represented. One-way ANOVA followed by Tukey’s post hoc test was used. Error bars represent ± SD, ** *p* < 0.001, and *** *p* < 0.0001.

To elucidate the post-translational regulation of MSX1 protein by DUBs, we carried out an unbiased genome-wide screening for DUBs that regulate MSX1 protein levels using our recently developed CRISPR/Cas9-based DUB knockout sgRNA kit [32]. To this end, we transfected Myc-MSX1 and Cas9 along with sgRNAs targeting 50 USP-subfamily genes individually. From our screening, the knockout of *USP11* and *USP49* showed low MSX1 protein levels, indicating that the depletion of these two DUBs resulted in reduced MSX1 protein (Figure 1a, Supplementary Figure S1). Among the two DUBs regulating MSX1 protein levels, the knockout of *USP11* showed the most marked reduction in MSX1 protein (Figure 1a). We cross-confirmed the effect of putative DUBs on MSX1 protein levels by designing two sgRNAs specifically at exon 1 and 2 for *USP11* (Supplementary Figure S2a) and at exon 1 for *USP49,* respectively. The sgRNA targeting *USP11* showed a higher reduction in MSX1 when compared to sgRNA targeting *USP49* in HEK293 cells (Figure 1b, Supplementary Figure S3a). Similarly, the depletion of USP11 showed a higher reduction in endogenous MSX1 protein when compared to sgRNA targeting *USP49* in hMSCs (Figure 1c, Supplementary Figure S3b).

### 2.2. USP11 Increases MSX1 Protein Level

**Figure 2:**
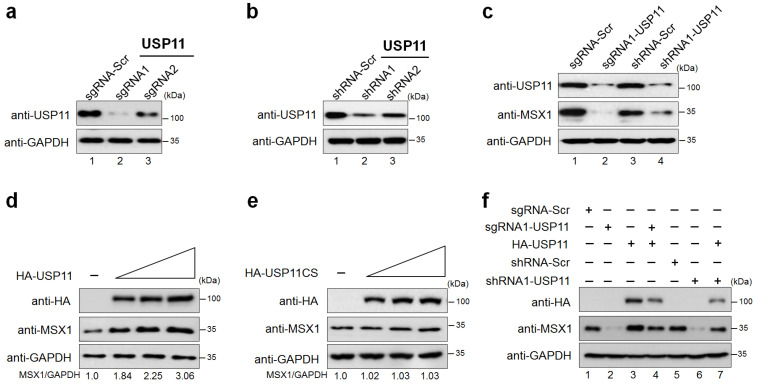
USP11 increases MSX1 protein level. (**a**) The efficiencies of sgRNA1 and sgRNA2 targeting the *USP11* gene and regulating endogenous MSX1 protein levels were determined in hMSCs. (**b**) The efficiencies of shRNA1 and shRNA2 targeting the *USP11* gene and regulating endogenous MSX1 protein levels were determined in hMSCs. (**c**) hMSCs were transfected with the best sgRNA (sgRNA1) and shRNA (shRNA1) targeting *USP11* for the evaluation of endogenous USP11 and MSX1 levels using the respective endogenous antibodies. (**d**) hMSCs were transfected with an increasing amount of HA-USP11 (0, 1, 2, and 3 µg), and the endogenous expression of MSX1 protein was analyzed using Western blot. The protein band intensities were estimated using ImageJ software with reference to the GAPDH control band (MSX1/GAPDH). (**e**) hMSCs were transfected with increasing amounts of catalytically inactive HA-USP11CS (0, 1, 2, and 3 µg), and the endogenous expression of MSX1 protein was analyzed using Western blot. The protein band intensities were estimated using ImageJ software with reference to the GAPDH control band (MSX1/GAPDH). (**f**) Reconstitution effect of USP11 on endogenous MSX1 in USP11-depleted cells. Protein expression was analyzed by Western blotting using the indicated antibodies. GAPDH was used as the loading control.

To validate our screening results, we transiently silenced *USP11* using both sgRNA and shRNA. Our results demonstrated that the protein expression of USP11 was significantly reduced by sgRNA1 when compared to sgRNA2 (Figure 2a, lane 2 vs. lane 3), which is in line with the high indel percentage obtained for sgRNA1 by T7E1 assay (Supplementary Figure S2b). The shRNA1 showed a higher reduction in USP11 expression than shRNA2 (Figure 2b, lane 2 vs. lane 3). Therefore, we used sgRNA1 (hereafter called sgRNA) and shRNA1 (hereafter called shRNA) targeting *USP11* in our further studies. The depletion of *USP11* using sgRNA and shRNA showed significant downregulation of endogenous (Figure 2c, lanes 2 and 4) as well exogenous MSX1 protein levels as compared to the mock control (Supplementary Figure S4a, lanes 3 and 5).

Next, we analyzed the effect of increasing the concentration of USP11 on endogenous MSX1 protein in hMSCs. As a result, we found that USP11 increased the expression of endogenous MSX1 protein in a dose-dependent manner (Figure 2d). In contrast, overexpression of USP11 catalytic mutant with a cysteine to serine mutation at position 318 (USP11CS) showed no impact on the endogenous MSX1 protein level (Figure 2e). Similarly, increasing concentrations of HA-USP11 in HEK293 cells also increased Myc-MSX1 protein in a dose-dependent manner (Supplementary Figure S4b). On the contrary, the USP11 catalytic mutant did not show any stabilization of exogenous MSX1 protein (Supplementary Figure S4c), indicating that USP11 stabilizes MSX1 protein through its deubiquitinating activity. Furthermore, we demonstrated that the reduced endogenous (Figure 2f, lanes 4 and 7) and exogenous (Supplementary Figure S5, lanes 4 and 7) MSX1 proteins were rescued when USP11-depleted cells were reconstituted with HA-USP11.

### 2.3. USP11 Interacts and Deubiquitinates MSX1

**Figure 3:**
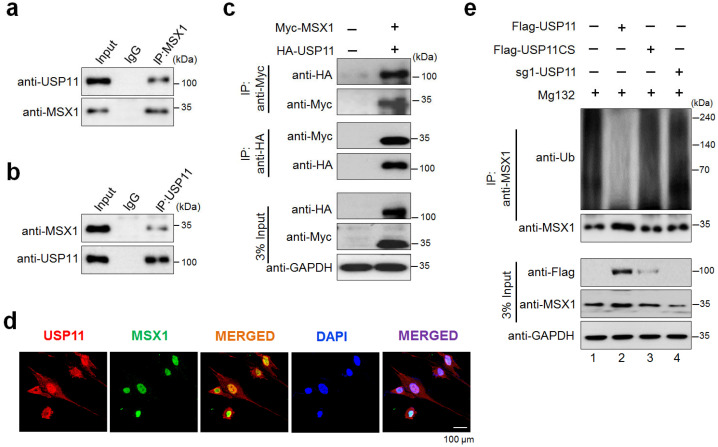
USP11 interacts and deubiquitinates MSX1 protein. (**a**,**b**) Interaction between endogenous USP11 and MSX1 proteins was evaluated in hMSCs. Cell lysates from hMSCs were immunoprecipitated with specific MSX1 (**a**) or USP11 antibodies (**b**) and immunoblotted with the indicated antibodies. (**c**) Myc-MSX1 and HA-USP11 were co-transfected in HEK293 cells. Samples were immunoprecipitated using anti-Myc or anti-HA antibodies and immunoblotted using the indicated antibodies. GAPDH was used as a loading control. (**d**) An immunofluorescence assay was performed to determine the localization of MSX1 and USP11 proteins. DAPI was used as a nuclear stain. (**e**) The endogenous deubiquitination of MSX1 was analyzed by transfecting hMSCs with Flag-USP11, Flag-USP11CS, and sgRNA targeting *USP11*, followed by immunoprecipitation with anti-MSX1 antibody and immunoblotting with anti-MSX1 or anti-ubiquitin antibodies. The cells were treated with MG132 for 6 h prior to harvest for all experiments.

We next sought to determine whether USP11 interacts with MSX1 protein within the cells. For this, we performed co-immunoprecipitation (co-IP) analysis in hMSCs using specific antibodies against endogenous USP11 or MSX1. We observed that USP11 co-precipitated with MSX1 and vice-versa (Figure 3a,b). Likewise, we cross-confirmed the interaction between USP11 and MSX1 by ectopically transfecting HA-USP11 and Myc-MSX1 in HEK293 cells. Our results showed that HA-USP11 co-immunoprecipitated with Myc-MSX1 and vice-versa (Figure 3c), indicating that USP11 interacts with MSX1 protein. Furthermore, the cellular localization behavior of MSX1 and USP11 proteins indicated that these two proteins were expressed in the nuclear region of hMSCs (Figure 3d).

To investigate the regulatory mechanism of USP11 on MSX1 protein, we checked whether the deubiquitinating activity of USP11 is required to prevent endogenous MSX1 proteolysis in hMSCs. Our results showed that USP11 overexpression remarkably reduced endogenous MSX1 ubiquitination in vivo (Figure 3e, lane 2), while Flag-USP11CS and sgRNA targeting *USP11* did not counteract the ubiquitination of endogenous MSX1 (Figure 3e, lane 3, 4), indicating that the deubiquitinating activity of USP11 prevents MSX1 protein degradation.

### 2.4. USP11 Enhances Osteogenic Transcriptional Factors in hMSCs

**Figure 4:**
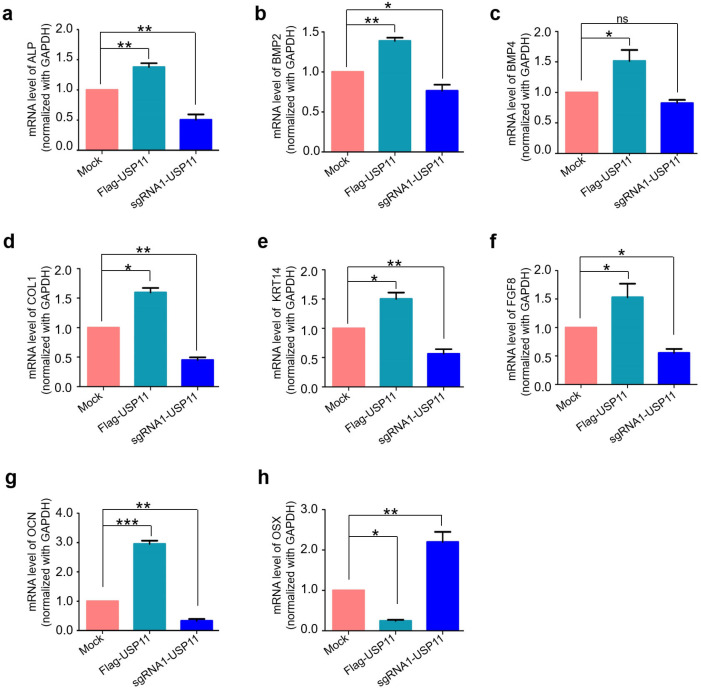
USP11 enhances osteogenic transcriptional factors in hMSCs. The mRNA levels of respective osteogenic markers (**a**) *ALP*, (**b**) *BMP2*, (**c**) *BMP4*, (**d**) *COL1*, (**e**) *KR14*, (**f**) *FGF8*, (**g**) *OCN*, and (**h**) *OSX* were quantified by qRT-PCR in hMSCs stably expressing Flag-USP11 and hMSCs with sgRNAs targeting *USP11* in comparison to a mock control. The average values from three independent experiments are shown. Error bars represent ± SD, ns= non significance, * *p* < 0.05, ** *p* < 0.001, and *** *p* < 0.0001 by ANOVA followed by Tukey’s post hoc test.

Next, we performed our functional analysis of the USP11-mediated regulation of osteogenic transcriptional factors in hMSCs which is a relevant cell type to study osteogenesis-related studies. The transcriptional expression pattern of osteogenesis-related transcription factors such as alkaline phosphatase (*ALP*), bone morphogenetic protein 2 (*BMP2*), bone morphogenetic protein 4 (*BMP4*), collagen 1 (*COL1*), Keratin 14 (*KRT14*), Fibroblast Growth Factor 8 (*FGF8*), osteocalcin (*OCN*; also known as *BGLAP*) and osterix (*OSX*) were evaluated by qRT-PCR analysis. To this end, we first generated overexpressed USP11 and USP11-depleted hMSCs and checked their expression using Western blot analysis (Supplementary Figure S6). The same cells were used for the qRT-PCR analysis of different osteogenesis-related transcriptional factors. Our results revealed that the overexpressed USP11 increased the activation of osteogenic differentiation-related markers up to 1.3- to 3-fold (Figure 4). The *OSX* showed a reduction in the transcriptional levels as compared to the mock cells (Figure 4h). Indeed, low transcriptional levels of *OSX* have been associated with the early stages of osteogenesis [33], which is in line with our results. In contrast, the USP11-depleted hMSCs significantly decreased the transcriptional levels of osteogenic differentiation-related markers (Figure 4). Overall, our results suggest that USP11 regulates the mRNA expression of osteogenesis-related transcriptional factors.

### 2.5. USP11 Enhances Osteogenic Activity in hMSCs

**Figure 5:**
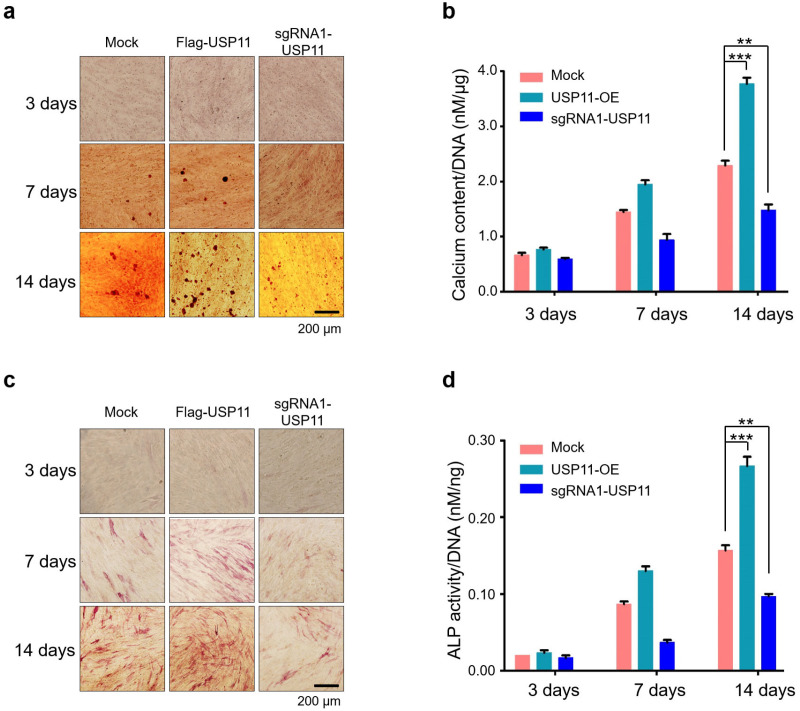
USP11 enhances osteogenic activity in hMSCs. hMSCs stably expressing USP11 or with sgRNA targeting, *USP11* and mock control hMSCs were incubated for 2 days in growth medium before the cultures became confluent. Thereafter, the cells were cultured in the osteogenic-induction medium. (**a**) Representative images of alizarin red staining were performed in each group at the indicated time points (days 3, 7, and 14). Scale bar: 200 µm. (**b**) The calcium content was quantified using the CPC method and normalized to the DNA content per well as measured on days 3, 7, and 14 to quantify the alizarin red staining. The data presented here are the mean ± SD of three independent experiments (** *p* < 0.001 and *** *p* < 0.0001 by ANOVA followed by Tukey’s post hoc test). (**c**) Representative images of cells from each group stained with alkaline phosphatase at the indicated time points (days 3, 7, and 14). Scale bar: 200 µm. (**d**) ALP activity was quantified and normalized to the DNA content obtained from each dataset on days 3, 7, and 14. The data presented here are the mean ± SD of three independent experiments (** *p* < 0.001, and *** *p* < 0.0001 by ANOVA followed by Tukey’s post hoc test).

hMSCs are multipotent stem cells that are found in the bone marrow and are important for the formation and repair of skeletal tissues, such as cartilage and bone. Under defined conditions, MSCs have the potential to differentiate into important lineages, such as osteocytes, chondrocytes, and adipocytes [34,35,36]. To investigate the effect of USP11 on osteogenic differentiation, we performed functional assays such as alizarin red staining (ALZ) and alkaline phosphatase (ALP) staining in hMSCs. For this, mock-hMSCs, USP11-overexpression hMSCs, and USP11-depleted hMSCs were cultured under an osteogenic induction medium for 14 days. The differentiating cells were subjected to ALZ and ALP staining at days 3, 7, and 14. ALZ staining showed mineralized nodule-like aggregates during the first week of differentiation, which eventually increased towards the end of the second week. However, USP11-depleted hMSCs showed significantly reduced osteogenic differentiation, which was evident by reduced nodule-like aggregates compared to the mock hMSCs (Figure 5a). Additionally, when compared to the control, the intracellular quantification of calcified colonies in USP11-depleted hMSCs displayed reduced mineralization (Figure 5b).

To further elucidate the functional relevance of USP11 on osteogenesis, we performed ALP staining, which is an early marker for osteogenic differentiation [37]. When compared to the mock-control, the USP11-depleted hMSCs showed significantly reduced ALP activity, while hMSCs stably overexpressing USP11 showed significantly higher ALP activity (Figure 5c). Colorimetric analysis of intracellular ALP activity in USP11-overexpressed and USP11-depleted hMSCs also showed similar results to the visual observation of ALP staining (Figure 5d), indicating that USP11 promotes the osteogenic differentiation of hMSCs.

## 3. Discussion

The *MSX* family gene is essential during embryogenesis and early development of limbs and craniofacial tissues, including teeth and bone [7,33]. MSX1 is transiently expressed in undifferentiated precursor cells, undergoes downregulation upon terminal differentiation, and exhibits a vital role in skeletal muscle development and bone remodeling [38,39]. Despite considerable effort towards understanding the spatio-temporal variations of MSX1 expression during the development of limbs, craniofacial glands, neurotubes, and other structures, the protein regulation of MSX1 has not received much attention [38,40,41,42]. However, there are few reports on PTMs of MSX1 suggesting its importance during early limb development. For instance, the cyclin-dependent kinase 1 (CDK1)-mediated phosphorylation of Msx1 at Ser136 is critical for enhancing Fgf9 and Fgf18 expression responsible for normal cell proliferation during early limb development. Depletion of Msx1/2 results in the downregulation of Fgf9 and Fgf18 expression and Erk1/2 phosphorylation, which leads to serious defects in limb development in mice [7].

The protein turnover of any target protein is regulated by ubiquitination and deubiquitination activities [43], which are critical for maintaining stem cell pluripotency and differentiation. The balanced control over ubiquitination and deubiquitination processes of stem cell transcription factors determines cell fate [43]. Ubiquitination of stem cell transcription factors by E3 ligases results in stem cell differentiation, while DUB activity prevents proteolysis and stabilizes stem cell transcription factors to maintain stemness [23,43,44].

Bone remodeling is a simultaneous process of bone resorption and deposition of bone components that involve the participation of three main cell lineages—osteoclasts, osteoblasts, and osteocytes [45]. An imbalance between the activities of osteoblasts and osteoclasts disrupts bone homeostasis, leading to skeletal disorders like osteoporosis, Paget’s disease, and rickets [46,47]. Several studies have unveiled the role of USP in regulating the functions of osteoblasts and osteoclasts [16,48,49]. E3 ligases Smurf1, Itch, and Wwp1 downregulate osteoblast differentiation via RUNX2 degradation [50,51,52,53,54,55,56]. In addition, several DUBs also influence bone remodeling by regulating the function of osteoclasts and osteoblasts [18,19,57]. Considering the importance of USP-mediated regulation of target proteins in osteogenesis, we wished to screen for potential DUBs regulating MSX1 protein levels and their molecular functions during bone formation.

In our study, we used a genome-wide DUB knockout sgRNA kit [32] to deplete entire USP sub-family genes to analyze the protein level of MSX1. From our screening system, we identified USP11 as a potent DUB regulating MSX protein level (Figure 1). USP11 has been previously reported to augment TGFβ signaling through deubiquitinating TGFβ receptor ALK5 [58]. Interestingly, USP4 and USP15, the paralogs of USP11 [57], are also associated with osteogenesis [59,60]; however, there have been no prior reports on USP11 and its functions related to bone remodeling to our knowledge. Thus, we investigated the functional consequences, particularly related to osteogenic differentiation of the USP11 interaction with MSX1 in hMSCs. We demonstrated that USP11 interacts and stabilizes MSX1 through its deubiquitinating activity (Figure 2 and Figure 3). Moreover, the depletion of USP11 imparted a significant reduction in several osteogenic transcriptional factors in hMSCs (Figure 4).

The activity of ALP during the osteogenic differentiation process indicates the differentiation of mesenchymal stromal cells into osteoblasts. [61]. ALP activity can be determined using colorimetric assays in which p-nitrophenyl phosphate, a phosphate substrate, is dephosphorylated by ALP [62]. ALP is a commonly used early marker of osteogenesis that is also known to reflect the degree of osteogenic differentiation [63]. Alizarin red S staining is a widely used stain for identifying calcium-containing osteocytes in differentiated osteoblast. Since calcium deposition is a late-stage osteogenic differentiation marker, we anticipate an increase in calcium content during the culture period. In this study, we showed that the depletion of USP11 resulted in impaired hMSCs osteogenic differentiation by ALP activity and ALZ staining, while the overexpression of USP11 increased osteogenic differentiation, which was evident by increased nodule-like aggregates and ALP activity when compared to the mock hMSCs (Figure 5). Collectively, our results demonstrate the novel role of USP11 in regulating MSX1 protein during osteogenic differentiation, which could facilitate the improvement of bone regeneration therapeutics.

## 4. Materials and Methods

### 4.1. Plasmids, sgRNA, and shRNA

The mammalian expression vector human Flag-MSX1 [64] was kindly provided by Prof. Kyoungsook Park (Sungkyunkwan University, Seoul, South Korea). The Flag-MSX1 was further subcloned into pcDNA 3.1 Myc-vector. Flag-HA-USP11 (#22566), pQFlag-USP11 WT puroR, pQHA-USP11 WT puroR (#46749), pQHA-USP11 CS puroR (#46750 and #46747), pQFlag-USP11 CS puroR (#46748), HA-tagged ubiquitin (#18712), and Cas9-2A-GFP (#44719) were purchased from Addgene, Watertown, Massachusetts, USA.

For DUB screening, we used a plasmid encoding Cas9-2a-mRFP-2a-PAC (puromycin N-acetyl-transferase, a puromycin resistance gene) and a plasmid encoding single guide RNA (sgRNA); both constructs were purchased from Toolgen (Seoul, Korea). The sgRNA target sequences were designed using bioinformatics tools (www.broadinstitute.org, accessed on 27 January 2020) and cloned into the vectors as previously described [65]. Retroviral vectors along with packaging plasmids were kindly provided by Prof. Chang-Hwan Park and short hairpin RNA (shRNA) lentiviral vector constructs along with the packaging plasmids were kindly provided by Prof. Chung Hee Yong (both from Hanyang University, Seoul, Korea). Target sequences for the sgRNA and shRNA are listed in Supplementary Tables S1 and S2, respectively.

### 4.2. Antibodies

Mouse monoclonal antibodies against Flag (Anti-DDDDK-tag, M185-3L, 1:1000; MBL Life Science), Myc (SC-40,1:1000), USP11 (SC-365528), ubiquitin (SC-8017, 1:1000), HA (SC-7392, 1:1000), GAPDH (SC-32233, 1:1000), and normal mouse IgG (SC-2025, 1:1000) were purchased from Santa Cruz Biotech, Dallas, TX, USA, unless indicated otherwise. MSX1 (H00004487-M11, 1:1000; Novus Biologicals, Littleton, CO, USA) and rabbit polyclonal antibodies against MSX1 (SC-15395, 1:500; Abcam, 1:100) and 488/594-conjugated secondary antibodies (Cat no. A21207, Cat no. A21203, 1:200) (Life Technologies, Carlsbad, CA, USA) were used. Protein A/G Plus Agarose beads (SC-2003, Santa Cruz Biotech, Dallas, TX, USA); MG132 (Cat no. S2619, Selleckchem, Houston, TX, USA), a proteasomal inhibitor; protease inhibitor cocktail (Cat. no. 11836153001; Roche, Basel, Switzerland), RIPA buffer (Cat. no. R2002; Biosesang, Seongnam-si, South Korea), Protein 5X sample buffer (Cat no. EBA-1052; Elpis biotech, Taejon, Korea); puromycin (Cat no. 12122530), dexamethasone (Cat no. D4902), β-glycerophosphate (Cat no. G9891), L-ascorbic acid (Cat no. A8960), Hoechst dye (Cat No. 33258, Sigma, St. Louis, MO, USA), and DAPI (Cat no. H-1200) were purchased from the noted manufacturers.

### 4.3. Cell Culture

Human embryonic kidney cells (HEK293) were cultured in DMEM (GIBCO BRL, Rockville, MD, USA) supplemented with 10% fetal bovine serum (FBS; GIBCO BRL, Rockville, MD, USA) and 1% penicillin and streptomycin (GIBCO BRL, Rockville, MD, USA) at 37 °C in a humidified atmosphere with 5% CO_2_. Human bone marrow-derived mesenchymal stem cells (hMSCs) were maintained in Mesenchymal Stem Cell Basal Medium (MSCBM; Cat. No. PT-3238, Cambrex BioScience, Bergen County, NJ, USA) supplemented with MSCGM SingleQuots (Cat. No. PT-4105, Cambrex BioScience, Bergen County, NJ, USA) and 1% penicillin and streptomycin (GIBCO, BRL, Rockville, MD, USA) at 37 °C in a humidified atmosphere with 5% CO_2_. The cells were passaged every 2–3 days depending on cell confluence.

### 4.4. Transient Transfection and Transduction

For transfection of HEK293 cells, polyethyleneimine was used (Polysciences, Warrington, PA, USA). hMSCs were transfected using Lipofectamine Stem or 3000 (Cat no. L3000001, Thermo Fisher Scientific, Waltham, MA, USA).

Retroviruses were produced by co-transfecting Flag-USP49, a pMD packaging plasmid containing Gag-Pol (pMD-gag.pol), and a VSV-G envelope plasmid (pMD-VSV-G) into HEK293 cells in a 3:2:1 ratio. Cell supernatants were harvested 48 h after transfection and were either used to infect cells or stored at −80 °C. Cells were infected at a low confluence for 6 h with retroviral supernatants diluted 1:1 with normal culture medium in the presence of 10 ng/mL of polybrene (Sigma Aldrich, St. Louis, MO, USA) to obtain stable cell lines of hMSCs expressing Flag-USP11. After 48 h of infection, the cells were placed under puromycin selection for 2 days and then passaged before use.

### 4.5. T7E1 Assay

T7E1 assays were performed as previously described [66]. After isolating genomic DNA using DNeasy Blood & Tissue kits (Qiagen, Hilden, Germany) according to the manufacturer’s instructions. The region of DNA containing the nuclease target site was PCR-amplified using hemi-nested primers. Amplicons were denatured by heating and annealed to form heteroduplex DNA, which was treated with 5 units of T7 endonuclease 1 (New England Biolabs, Ipswich, MA, USA) for 15 to 20 min at 37 °C and analyzed using 2% agarose gel electrophoresis. Mutation frequencies were calculated based on band intensity using ImageJ software and the following equation: mutation frequency (%) = 100 × (1 − [1 − fraction cleaved] 1/2), where the fraction cleaved was the total relative density of the cleavage bands divided by the sum of the relative density of the cleavage and uncut bands. The oligonucleotide sequences used to produce the PCR amplicon for the T7E1 assay are listed in Supplementary Table S3. The amplicon size of the *USP49* gene and expected cleavage sizes after the T7E1 assay are summarized in Supplementary Table S4.

### 4.6. Immunofluorescence

For the co-localization studies, hMSCs were seeded in a 24-well plate and incubated at 37 °C in a humidified atmosphere with 5% CO_2_ for 36 h. After a PBS wash, the cells were fixed for 15 min in 4% PFA and permeabilized with 0.1% Triton X-100 in PBS for 10 min. The slides were incubated with the respective primary antibodies at 4 °C overnight. After washing them with PBS, we incubated the slides with 1 μg/mL Alexa Fluor 488/594-conjugated secondary antibodies and counterstained them using VECTASHIELD anti-fade mounting medium with DAPI and To-Pro for the cell and tissue sections, respectively. The stained immunofluorescence images were captured using a confocal laser microscope (LSM510META Ver. 3.2; Carl Zeiss, Oberkochen, Germany or DMi8; Leica, Wetzlar, Germany).

### 4.7. Immunoprecipitation

After 48 h of transfection, cells were harvested and lysed for 20 min in RIPA buffer containing 150 mM sodium chloride, 1% Triton X-100, 1% sodium deoxycholate, 0.1% SDS, 50 mM Tris-HCl (pH 7.5), 2 mM EDTA along with protease inhibitor cocktail. Then, 2–3 mg of cell lysates were immunoprecipitated with the respective antibodies at 4 °C overnight and combined with 25 μL of protein agarose beads at 4 °C for 2–3 h. The beads were washed with RIPA buffer and eluted in 5X SDS sample loading buffer containing 4% SDS, 20% glycerol, 10% 2-mercaptoethanol, 0.004% bromophenol blue, and 0.125 M Tris-HCl (pH 6.8) and boiled at 95–100 °C for 5 min. The samples were then loaded onto SDS-PAGE gels and analyzed by Western blotting using a ChemiDoc Imaging System, Bio-Rad Laboratories, Hercules, CA, USA.

### 4.8. Deubiquitination Assay

The DUB activity of USP11 and its effect on endogenous MSX1 was examined in hMSCs. After 48 h, cells were treated with MG132 (5 µM/mL for 6 h) and harvested, followed by immunoprecipitation and Western blotting with the indicated antibodies.

### 4.9. Real-Time PCR

Total RNA was isolated using Trizol reagent (Favorgen, Kaohsiung, Taiwan). RNA pellets were suspended in 30 μL of nuclease-free water, after which the RNA concentration was determined. Total mRNAs were reverse transcribed into cDNA using the SuperScript III First-Strand Synthesis System (Life Technologies, Carlsbad, CA, USA) with an oligo dT primer. Quantitative PCR was performed using Fast SYBR Green master mix (Life Technologies, Carlsbad, CA, USA) and the StepOnePlus Real-Time PCR System (Life Technologies, Carlsbad, CA, USA). Real-time primers were designed individually for osteogenic-specific genes, listed in Supplementary Table S5. A GAPDH primer set was used for normalization. After DNA amplification, expression levels were determined with GraphPad Prism 9 (GraphPad Software, San Diego, CA, USA).

### 4.10. Osteogenic Differentiation of hMSCs

At Passage 3, hMSCs were examined for their osteogenic potential. hMSCs were seeded onto 6-well plates and then cultured with osteogenesis induction medium (basal medium with 0.1 μM dexamethasone, 10 mM β-glycerophosphate, and 60 μM L-ascorbic acid) and 1% penicillin and streptomycin for 14 days. The cells were grown for two weeks, with the induction medium changing every other day.

### 4.11. Alizarin Red Staining

hMSCs mineralization in the control and experimental groups was examined using alizarin red staining on days 3, 7, and 14. Osteogenic cultures were washed twice with PBS and fixed in 4% PFA for 15 min, and then alizarin red (Cat no. A5533, Sigma, St. Louis, MO, USA) staining was performed according to the manufacturer’s protocol. Using phase-contrast microscopy, red stains indicating calcium deposition were observed. To determine the calcium content, 1 mL of 10% cetylpyridinium chloride (CPC) (Sigma, St. Louis, MO, USA) was added to both control and experimental samples, and stains were eluted after 20–30 min. 100 μL of the eluted stain in CPC was added to a 96-well plate, and the reading was taken at 550 nm using a spectrophotometer. A standard curve was prepared using alizarin red stain and CPC. The molar equivalent of calcium was used to express calcium deposition. In an alizarin red S-calcium complex, one mole of alizarin red binds to two moles of calcium.

### 4.12. Alkaline Phosphatase Activity

Osteogenic cultures were washed twice with PBS and fixed in 4% PFA for 15 min. ALP staining (Cat no. 86R-1KT, Sigma Aldrich, St. Louis, MO, USA) was performed on days 3, 7, and 14 according to the manufacturer’s protocol. Intracellular activity of ALP was measured at different time points using the ALP colorimetric assay kit (Abcam plc, Cambridge, UK) that used p-nitrophenyl phosphate (pNPP) as a phosphatase substrate. Briefly, cells from all the groups were washed with PBS and lysed with ALP assay buffer. Thereafter, a total of 80 µL of the cell lysate was added to 50 μL of pNPP in a 96-well plate, and the samples were shielded from direct light for 1 h at room temperature. Thereafter, 20 µL of stop solution (3N NaOH) was added to the wells, and the plate was read at 415 nm using a spectrophotometer.

### 4.13. DNA Content for Alizarin Red Staining and ALP Assays

DNA content in the cell lysates prepared for the alizarin red staining and ALP assays was measured using the fluorescent dye bisBenzimide (Hoechst). Briefly, 50 μL of each cell lysate was added to separate 1.5 mL tubes containing 50 μL of assay buffer and 100 μL of Hoechst dye. A 0.1 μg/mL solution was used for the 3-day time point, and a 1 μg/mL solution was used for the 7 and 14 days. The plate was incubated away from light for 10 min and then subjected to excitation at 350 nm; emissions were monitored at 450 nm with an EnVision system (PerkinElmer, Waltham, MA, USA).

### 4.14. Statistical Analysis

All results are presented as the mean and standard deviation of at least three independent experiments (unless otherwise stated in the Figure legend). Comparisons between two groups were analyzed using the student’s *t*-test. Experiments involving three or more groups were analyzed by one-way or two-way analysis of variance (ANOVA) followed by Tukey’s post hoc test. A *p* value < 0.05 was considered statistically significant. All statistical analyses were performed in GraphPad Prism 9 software.

## Data Availability

The datasets used and/or analyzed during the current study are available from the corresponding author on reasonable request.

## Supplementary Materials

The following supporting information can be downloaded at: https://www.mdpi.com/article/10.3390/ijms23020856/s1.

## Abbreviations

ALP	Alkaline phosphatase
BMPs	Bone morphogenetic proteins
BSA	Bovine serum albumin
CHX	Cycloheximide
COL1	Collagen I
CRISPR	Clustered regularly interspaced short palindromic repeats
DSB	Double-strand break
DUBs	Deubiquitinating enzymes
FGF	Fibroblast growth factor
HEK293	human embryonic kidney cells
hMSCs	human Mesenchymal stem cells
IP	Immunoprecipitation
KRT14	Keratin 14
MSX1	Msh homeobox 1
OCN	Osteocalcin
OSX	Osterix
PAGE	Polyacrylamide gel electrophoresis
PAM	Protospacer adjacent motif
PTM	post-translational modification,
RUNX2	Runt-related transcription factor 2
SDS	Sodium dodecyl sulfate
sgRNA	Single guide RNA
shRNA	Short hairpin RNA
TGFβ	Transforming growth factor β
T7E1	T7 Endonuclease I
UCH	Ubiquitin carboxyl-terminal hydrolase
USP	Ubiquitin-specific protease
