# Supplementary material for: Genome-Wide CRISPR/Cas9-Based Screening for Deubiquitinase Subfamily Identifies Ubiquitin-Specific Protease 11 as a Novel Regulator of Osteogenic Differentiation
Source: Int J Mol Sci. 2022 Jan 13;23(2):856. doi: 10.3390/ijms23020856 (PMC8778097; doi:10.3390/ijms23020856)

Supplementary Materials

Genome-wide CRISPR/Cas9-based screening for deubiquitinase subfamily identifies Ubiquitin-specific protease 11 as a novel regulator of osteogenic differentiation

Kamini Kaushal[1†], Apoorvi Tyagi[1†], Janardhan Keshav Karapurkar[1], Eun-Jung Kim[2], Parthasaradhireddy Tanguturi[1], Kye-Seong Kim[1,3*], Han-Sung Jung[2*], and Suresh Ramakrishna[1,3*]

[1]Graduate School of Biomedical Science and Engineering, Hanyang University, Seoul, 04763, South Korea

[2]Division in Anatomy and Developmental Biology, Department of Oral Biology, Taste Research Center, Oral Science Research Center, BK21 FOUR Project, Yonsei University College of Dentistry, Seoul, 03722, South Korea

[3]College of Medicine, Hanyang University, Seoul, 04763, South Korea

[†]These authors contributed equally: Kamini Kaushal and Apoorvi Tyagi

***Corresponding authors**

SR (E-mail: suri28@hanyang.ac.kr, suresh.ramakris@gmail.com);

HSJ (E-mail: hsj8076@gmail.com)

KS (E-mail: ks66kim@hanyang.ac.kr);

Supplementary Figures:

Figure S1. Expression of Myc-MSX1 normalized with respect to GAPDH.

Figure S2. Comparison of sgRNA efficiencies in targeting the *USP11* gene in hMSCs.

Figure S3. The triplicate images for the immunoblots represented as graphs in the main figure no. 1b and 1c.

Figure S4. USP11 increases MSX1 protein level.

Figure S5. USP11 reconstitution in USP11 depleted HEK293 cells.

Figure S6. Protein expression of transduced USP11 in hMSCs.

Supplementary Tables

Table S1. Target sequences used for sgRNA plasmid construction**.**

Table S2. Target sequences used for shRNA plasmid construction**.**

Table S3. Oligonucleotide sequences used to get PCR amplicon for T7E1 assay.

Table S4. PCR amplicon and cleavage sizes after T7E1 assay.

Table S5. Primers used for qRT-PCR.

Figure S7: Uncropped blots of main figures are after tables section.

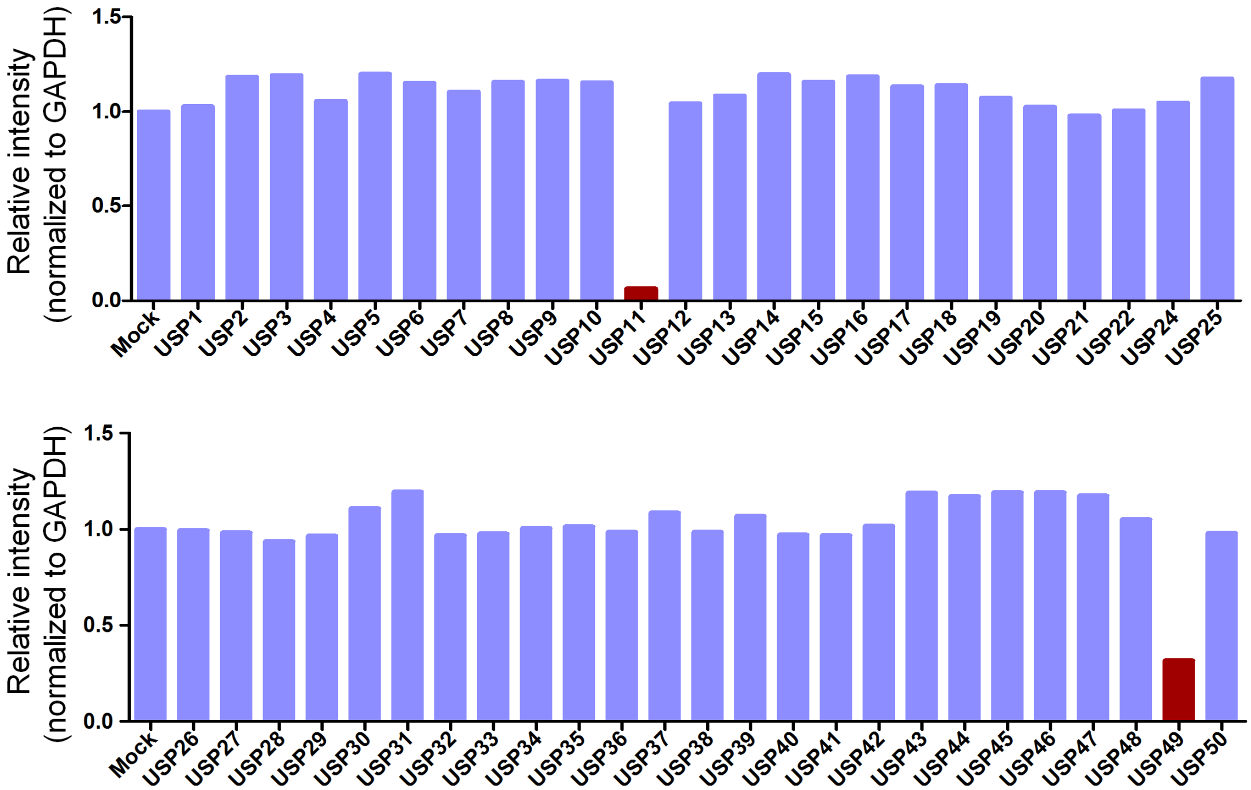

Figure S1: Expression of Myc-MSX1 normalized with respect to GAPDH. The protein band intensities were estimated using ImageJ software normalized to GAPDH control band for each individual sgRNA (Myc-MSX1/GAPDH). The loss of USPs leading to the downregulation of MSX1 protein level is marked in brown.

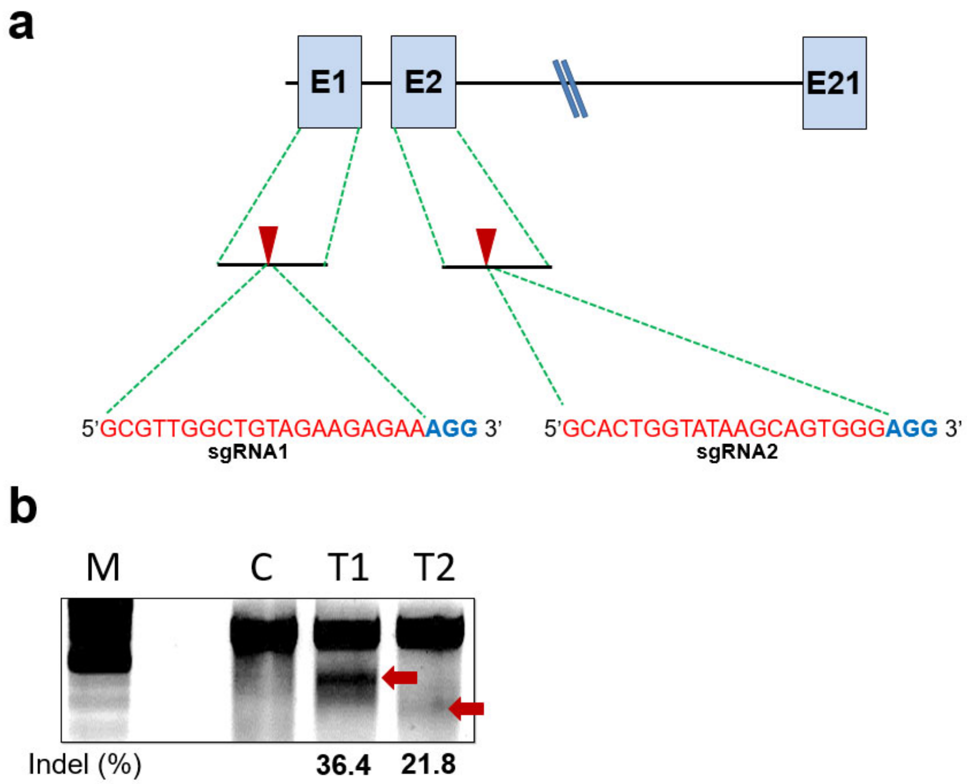

Figure S2. Comparison of sgRNA efficiencies in targeting the *USP11* gene.

(a) Schematic representation of the RNA-guided engineered nuclease (RGEN) targeting the human *USP11* gene via the designed sgRNA1 (T1), sgRNA2 (T2), targeting sequences in exon 1 and exon 2 respectively. The blue boxes represent exons. Red arrowheads indicate the positions of sgRNAs that target the top strand. PAM sequences are presented in the bold blue font. (b) The cleavage efficiency of sgRNA1 (T1), sgRNA2 (T2) was determined by the T7E1 assay after transfection with plasmids encoding Cas9 and sgRNA. Non targeting sgRNA transfected cells were used as control (C). The size marker (M) is shown. The red arrow indicates the expected position of the cleaved DNA

bands. The numbers at the bottom of the gel indicate the mutation percentages measured by band intensity using ImageJ software.

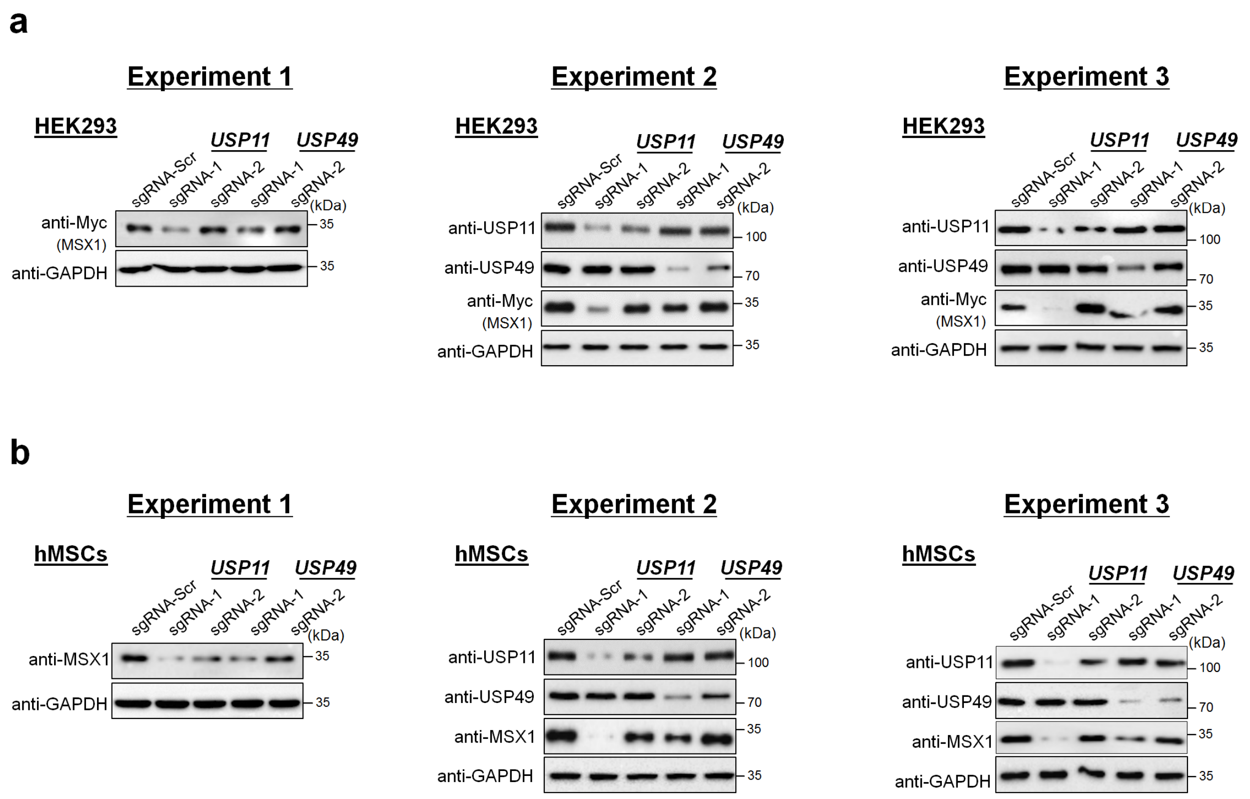

Figure S3. The triplicate images for the immunoblots represented as graphs in the main figure 1b and 1c.

(a) HEK293 cells were transiently transfected with sgRNA1 and sgRNA2 targeting either USP11 or USP49, along with ectopically expressed Myc-MSX1 to check exogenous MSX1 protein levels.

(b) hMSCs were transiently transfected with sgRNA1 and sgRNA2 targeting either USP11 or USP49 to check endogenous MSX1 protein levels.

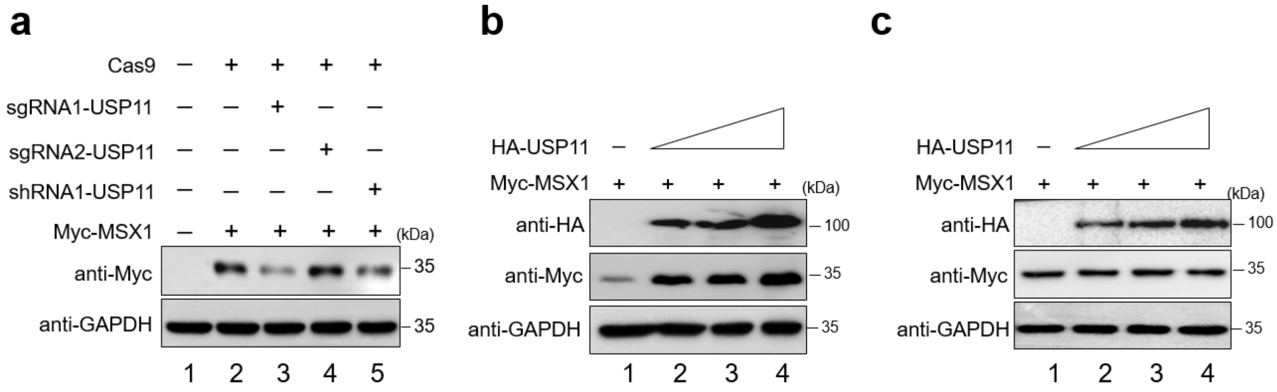

Figure S4. USP11 increases MSX1 protein level.

(a) HEK293 cells were transiently transfected with sgRNA1, sgRNA2, and shRNA1, which all targeted *USP11*, along with ectopically expressed Myc-MSX1 to check exogenous MSX1 protein levels. (b) HEK293 cells were transfected with Myc-MSX1 and increasing concentrations of HA-USP11. (c) HEK293 cells were transfected with Myc-MSX1 and increasing concentrations of HA-USP11CS.

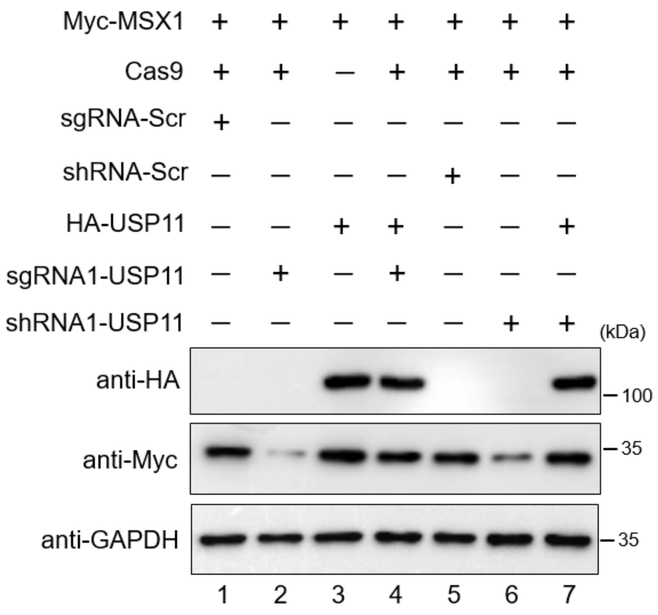

Figure S5. USP11 reconstitution in USP11 depleted HEK293 cells.

The rescue of Myc-MSX1 protein mediated by USP11 was analyzed by reconstitution with HA-USP11. Protein expression was detected through the indicated antibodies and analyzed by Western blotting. GAPDH were used as a loading control.

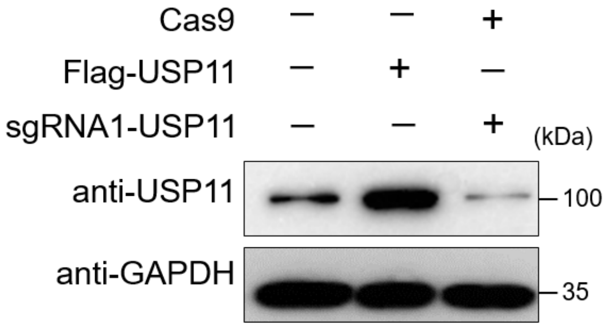

Figure S6. Protein expression of USP11 in hMSCs.

Lane 1 is Mock, Lane 2 is overexpression USP11 and Lane 3 is depleted USP11.

Table S1. Target sequences used for sgRNA plasmid construction.

USP11	*Homo sapiens*	sgRNA1	GCGTTGGCTGTAGAAGAGAA
		sgRNA2	GCACTGGTATAAGCAGTGGG

Table S2. Target sequences used for shRNA plasmid construction.

USP11	*Homo sapiens*	shRNA1	CCGTGATGATATCTTCGTCTA
		shRNA2	CGGCACAATGATTTGGGCAAA

Table S3. Oligonucleotide sequences used to get PCR amplicon for T7E1 assay.

USP11	*Homo sapiens*	sgRNA1	FP	TCAGCGTCCCCATTGTTACC
			FP1	GGGTCTCTGGAGGTGGAAAC
			RP	ATCGCAACGTCTGGAAAAGG
			RP1	GCGAAAGCTGGTGAGGCTGG
		sgRNA2	FP	AAATTAGCTGCAGATGGCACA
			FP1	TGTTGGTTTGTTTGCCATTG
			RP	ACGCAAGGTATAATGGATGGGG

Table S4. PCR amplicon and cleavage sizes after T7E1 assay.

USP11 *Homo sapiens*	sgRNA1	756	381+375
	sgRNA2	681	286+395

Table S5. Primers used for qRT-PCR.

OCN	FP: 5′-GCCTCCTGAAAGCCGATGT-3′
	RP: 5′-AAGAGACCCAGGCGCTACCT-3′
OSX	FP: 5′-GCCAGAAGCTGTGAAACCTC-3′
	RP: 5′-GCTGCAAGCTCTCCATAACC-3′
BMP4	FP: 5′-AGCATGTCAGGATTAGCCGA-3′
	RP: 5′-TGGAGATGGCACTCAGTTCA-3′
BMP2	FP: 5′- ATGGATTCGTGGTGGAAGTG-3′
	RP: 5′-GGCCAAAAGTTACTAGCAATGG-3′
FGF8	FP: 5′-GGCCTCTACATCTGCATGAAC-3′
	RP: 5′-CCCTCGTACTTGGCATTCTG -3′
COL1	FP: 5′-AAGGACAAGAGGCACGTCTG-3′
	RP: 5′-CGCTGTTCTTGCAGTGGTAG-3′
ALP	FP: 5′-ACGGGAAGAATCTGGTGCAG-3′
	RP: 5′-TGTGGAGTCTCGGTGGATCT-3′
KR14	FP: 5′-AGACCATTGAGGACCTGAGGA-3′

	RP: 5′-TGATGTCGGCTTCCACACTC-3′
GAPDH	FP: 5′-CATGTTCGTCATGGGTGTGAACCA-3′
	RP: 5′-AGTGATGGCATGGACTGTGGTCAT-3′

Figure S7: Uncropped blots from main figures

Uncropped blots of Figure 1

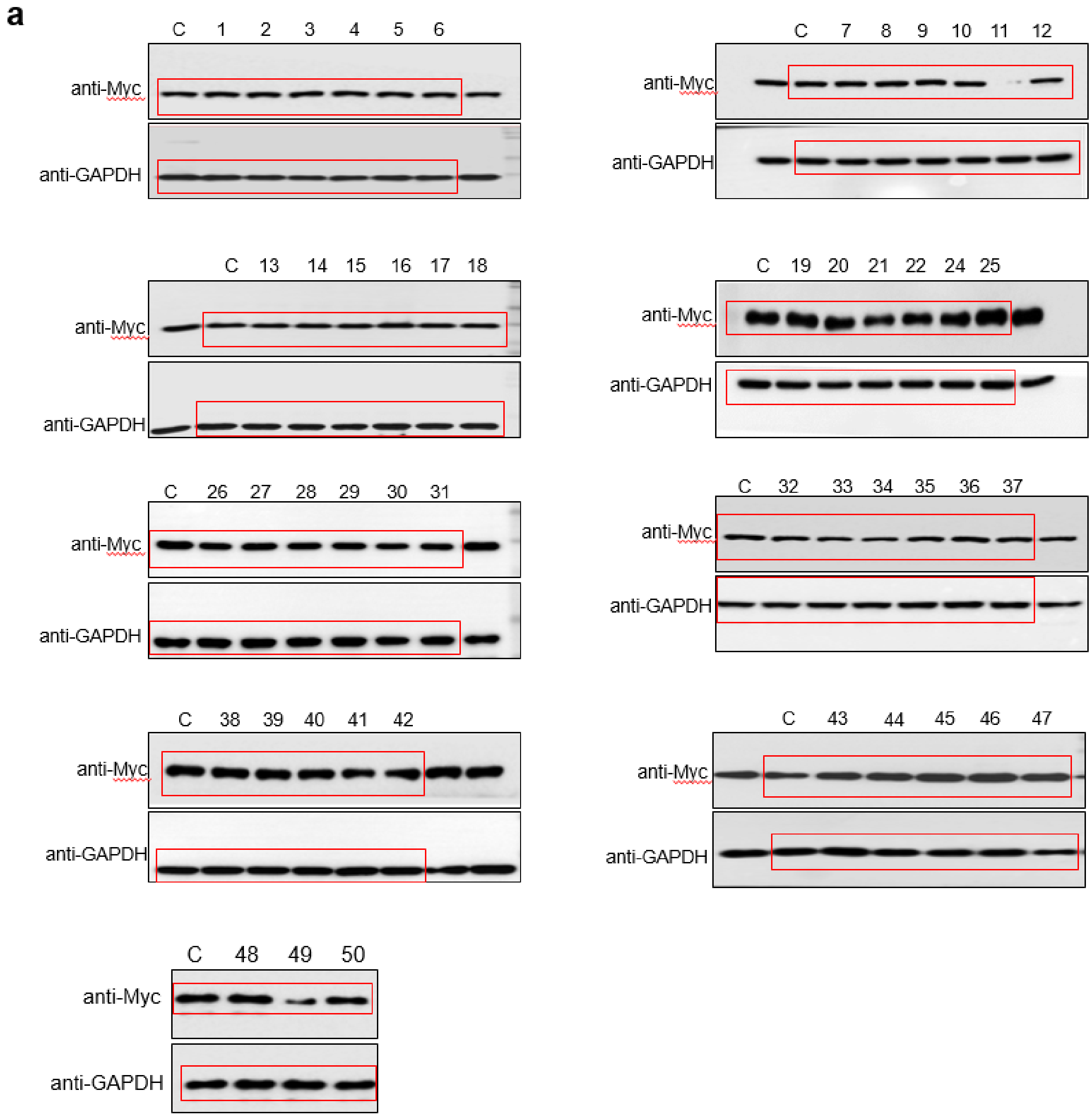

Uncropped blots of Figure 1

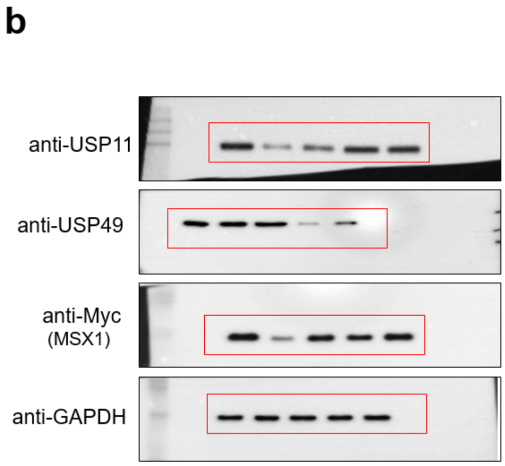

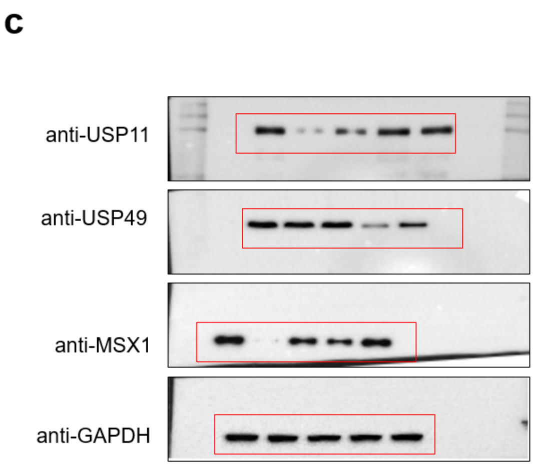

Uncropped blots of Figure 2

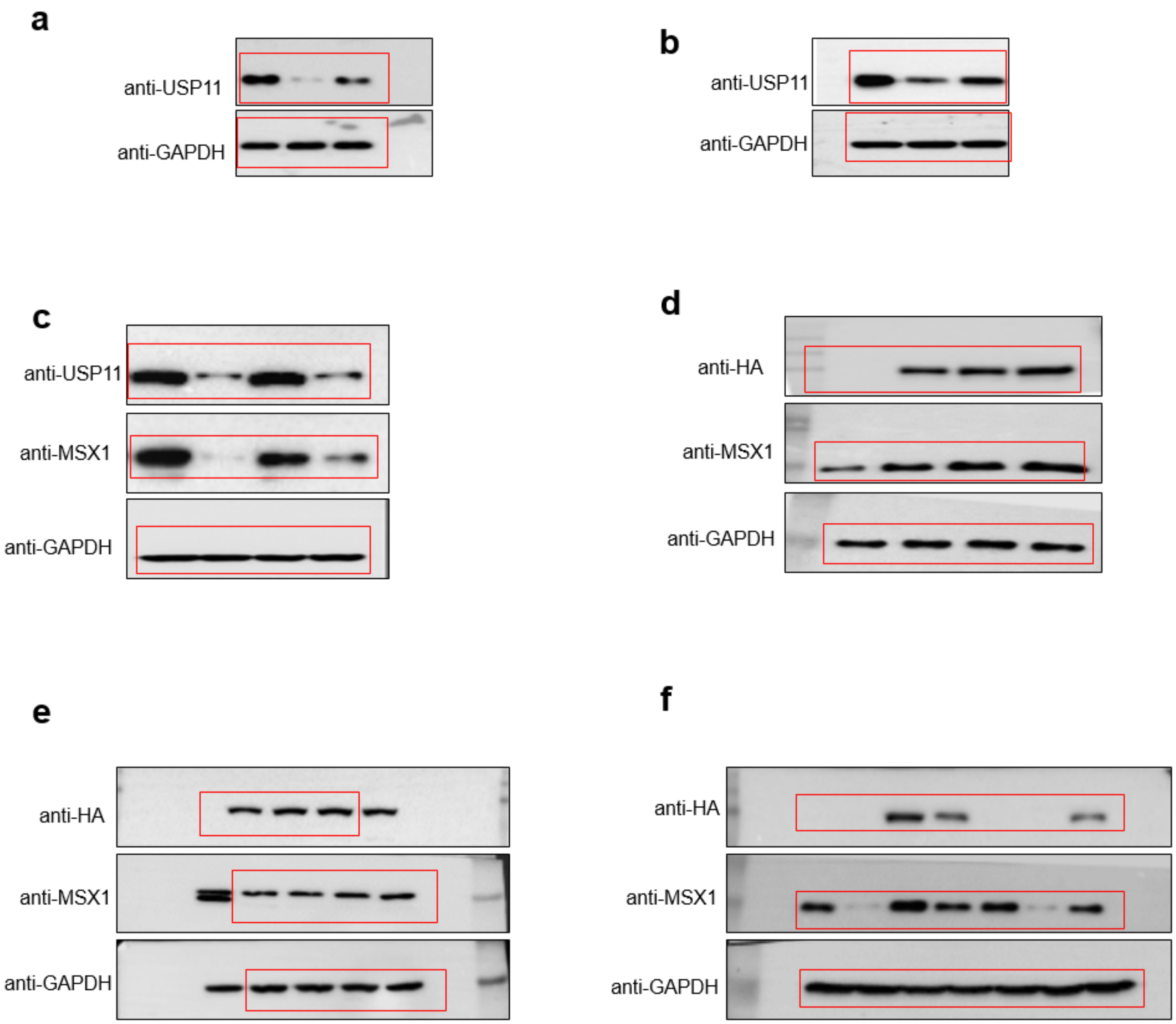

Uncropped blots of Figure 3

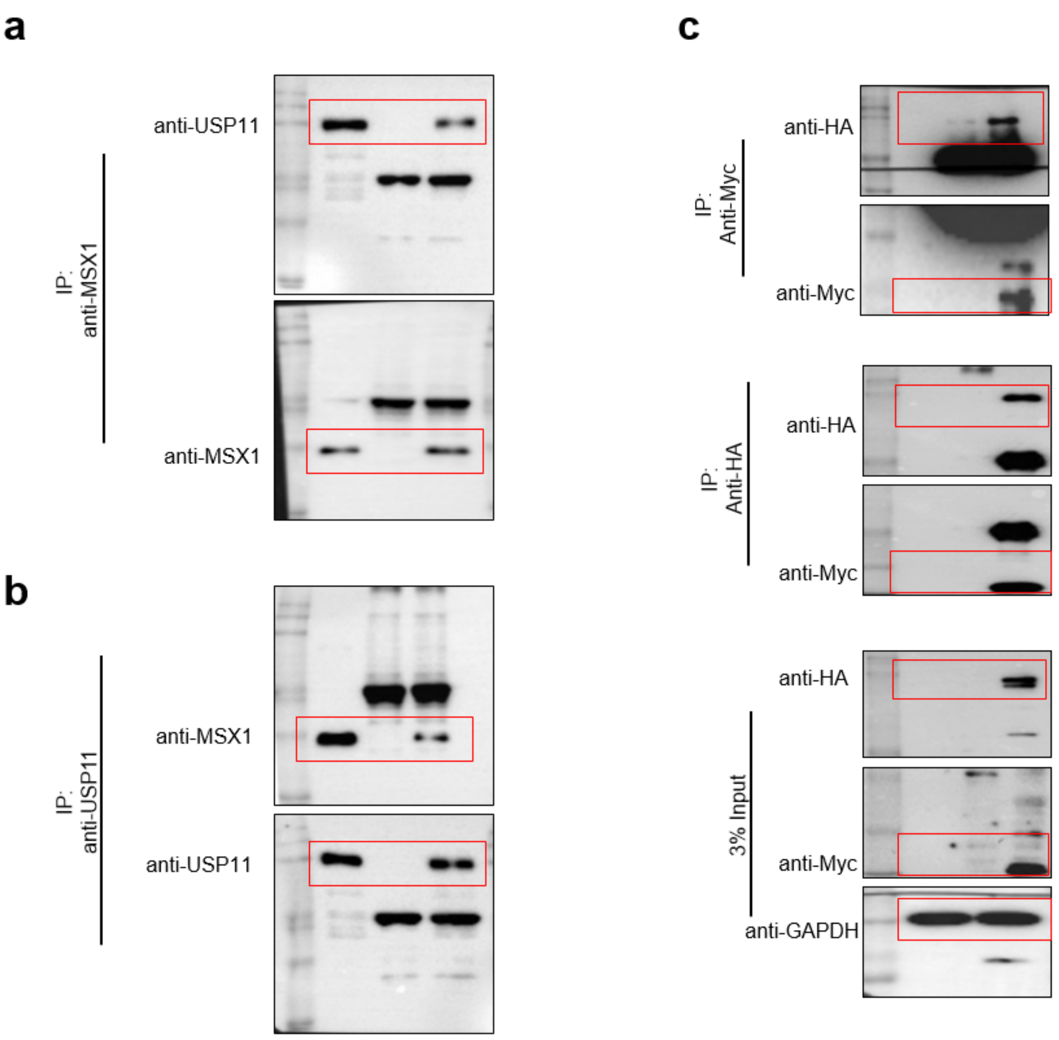

Uncropped blots of Figure 3

e

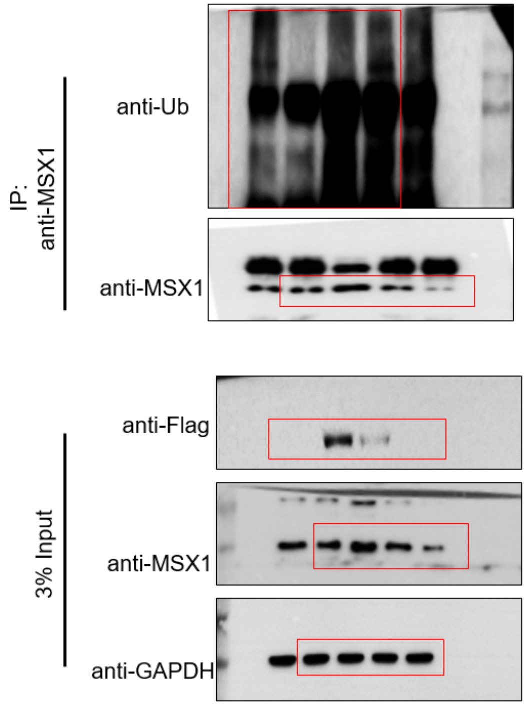

Supplement: Supplementary file 1 [file ijms-23-00856-s001.zip › ijms-1531239-supplementary.pdf]
